# Postoperative Outcomes of One-Step Implant-Based Breast and Ovarian Surgery in High-Penetrance Gene Mutation: A Single-Center Experience

**DOI:** 10.3390/jcm14061784

**Published:** 2025-03-07

**Authors:** Buse Irem Koc, Sevket Barıs Morkavuk, Simay Akyuz, Guzin Aygun, Ozhan Ozdemir, Mehmet Ali Gulcelik

**Affiliations:** 1 Department of General Surgery, Gulhane Education and Research Hospital Ankara, University of Health Sciences, 06010 Ankara, Turkey; guzinbulbuloglu@gmail.com; 2 Department of Surgical Oncology, Gulhane School of Medicine, University of Health Sciences, 06018 Ankara, Turkey; sevketbaris.morkavuk@sbu.edu.tr (S.B.M.); mgulcelik@yahoo.com (M.A.G.); 3 Faculty of Nursing, Gulhane School of Nursing, University of Health Sciences, 06018 Ankara, Turkey; simayakyuz@gmail.com; 4 Department of Gynecology and Obstetrics, Gulhane School of Medicine, University of Health Sciences, 06018 Ankara, Turkey; ozhan.ozdemir@saglik.gov.tr

**Keywords:** high-penetrance gene mutations, risk-reducing surgery, salpingo-oophorectomy, skin sparing mastectomy, BRCA1-BRCA2, implant complications, chemotherapy, radiotherapy

## Abstract

**Background/Objectives**: This study was designed to evaluate skin-sparing mastectomy with implant reconstruction complication rates in patients operated on due to high penetrant gene profile. All patients went to skin-sparing mastectomy with implant reconstruction and risk-reducing salpingo-oophorectomy. The effect of radiotherapy and chemotherapy on wound healing is a frequently discussed topic in the literature. However, studies on the effect of these on patients undergoing implant-based reconstruction are rare. In our clinic, two surgeries are performed under the same anesthesia and it is aimed to investigate the effect of this situation on complications in this rare patient group. In this retrospective study, we report our clinical experience regarding complication rates due to these factors among the high penetrant gene group. **Methods**: Between June 2022 and June 2024, 61 patients were grouped according to demographic data. Post-operative complications were defined as any of the following: major complications which were active bleeding or wound dehiscence; minor complications which were hematoma, seroma, surgical-site infection, <20% skin or nipple necrosis, and reoperation due to wound dehiscence or any other complication. Patients were compared in terms of complications according to whether they received previous radiotherapy (RT), neoadjuvant chemotherapy (CT), or underwent skin-reducing mammoplasty. **Results**: Patients receiving neoadjuvant chemotherapy, patients receiving preoperative RT, and patients undergoing skin-reducing mastectomy were compared in terms of major and minor complications. While neoadjuvant CT and preoperative RT only increased the risk of seroma, it was found that skin-reducing mastectomy had no significant effect on complication rates. **Conclusions**: Skin-sparing mastectomy with implant reconstruction and risk-reducing salphingo-oophorectomy is a comprehensive operation method in this patient group. Complication control can be achieved by performing two surgeries in a single anesthesia period, using the spy immunofluorescence device for vascularization control, and performing all surgeries with the same experienced team.

## 1. Introduction

Breast cancer is one of the most common cancers among women, affecting one in eight women. Hereditary cancers account for 5% of breast cancers and high penetrant genes are the leading ones. Patients with high-penetrance gene mutations have an increased lifetime risk of developing not only breast but also ovarian cancer. BRCA1/2 genes and other high-penetrance genes, such as TP53, CDH1, PTEN, or STK11 and low to medium-penetrance genes, such as ATM, CHEK2, BRIP1, or PALB2 are risk factors for the development of breast cancer [1,2]. Overall, BRCA1/2 mutations are responsible for approximately 5% of breast cancer cases [3]. According to the National Comprehensive Cancer Network (NCCN), the risk-reducing salpingo-oophorectomy (RRSO) and the risk-reducing bilateral mastectomy (RRBM) can decrease the lifetime risk of developing ovarian cancer by more than 95% and breast cancer by 85–90% in patients carrying BRCA1/2 mutations [4,5]. Pathogenic mutations in other high-penetrance genes (i.e., TP53, PTEN, CDH1, NGO11, or PALB2) other than BRCA1/2 increase the risk of breast cancer by more than 4-fold and are responsible for 25% of all hereditary breast cancers [6]. BRCA carriers are advised to undergo risk-reducing mastectomy (RRM) and risk-reducing oophorectomy after completing their childbearing period. For patients carrying PALB2, ATM, or CHEK2 mutations although the NCCN Guidelines^®^ (v.3.2019) warn that the evidence is insufficient to recommend RRM in patient groups with a family history of risk-reducing mastectomy is preferred for this patient group [7].

Neoadjuvant chemotherapy (NACT) facilitates breast reconstruction by reducing tumor size in locally advanced cancers. While NACT has beneficial effects, it is also thought to potentially have negative impacts on wound healing. Recent large-scale studies have shown that NACT does not significantly affect wound healing. There are several studies in the literature involving large patient populations on this topic. However, the only patient group not included in these studies is those who underwent immediate implant reconstruction following mastectomy [8,9]. This study is one of the few in the literature investigating neoadjuvant chemotherapy in patients who received implants immediately after mastectomy. In our country, genetic test results typically take up to two to three months to be available. As a result, some patients diagnosed with breast cancer undergo breast-conserving surgery before receiving the genetic test results. Breast-conserving surgery followed by adjuvant radiotherapy is a type of oncological surgery that is considered a safe option. However, in patients who require mastectomy after genetic test results, previous radiotherapy might also cause problems in terms of wound healing. It is reported in the literature that radiotherapy has negative effects on wound healing [10]. In comparison, skin-reducing mammoplasty is a risky method in terms of wound healing [11]. As such, questions are raised about whether complication rates increase when skin-reducing mammoplasty and skin-sparing mastectomy with implant reconstruction are used together. Breast surgery is a surgery with a wide spectrum of methods with developing oncoplastic techniques. Nowadays, the type of surgery can be determined with USG, MR, and especially 3D imaging in the preoperative period. We treated all patients in our study with bilateral skin-sparing subcutaneous mastectomy and subpectoral implant. [12].

Neoadjuvant chemotherapy due to locally advanced disease, previous radiotherapy to the chest wall, and skin-reducing mastectomy might increase wound healing and complications. In this study, we investigated the complication rates of neoadjuvant chemotherapy, previous radiotherapy, and skin-reducing procedures in patients who were operated on because of genetic test results. The high-penetrance gene mutation patient group is a group of patients who have undergone multiple operations for breast and ovarian surgery. In our study, the effect of performing two operations at the same time and in collaboration with a single experienced team on the risks of breast surgery is discussed. In this context, patients were compared over radiotherapy and chemotherapy groups, which potentially impair wound healing.

## 2. Materials and Methods

### 2.1. Study Design and Inclusion and Exclusion Criteria

Between June 2022, and June 2024, 85 patients who underwent surgery at the Health Sciences University, Gülhane Training and Research Hospital due to high-penetrance gene profile were enrolled. We included patients between the ages of 18 and 60 with high-penetrance gene mutations as a result of genetic testing due to age, family history, triple negative, and bilateral breast cancer. All patients underwent skin-sparing mastectomy with implant reconstruction and risk-reducing salpingo-oophorectomy. Patients who had undergone breast-conserving surgery as definitive surgery, unilateral skin-sparing mastectomy with implant reconstruction, bilateral mastectomy without implant reconstruction, and men were excluded. Patients who smoked were also excluded from the study to avoid bias in complication rates. The remaining 61 patients were grouped according to demographic data and compared in terms of complication rates. The name of the ethics committee we received approval for the study is Gülhane Scientific Research Council and the approval code is 2024/561. The date of acceptance is 10 December 2024.

In total, 24 patients had BRCA1 mutations, 28 had BRCA2 mutations, and 9 had other high-penetrance gene mutations. Patients were grouped by age, body mass index (BMI), related comorbidity (i.e., has an effect on wound healing process, such as diabetes mellitus and medical conditions requiring the use of steroids), hormone receptor condition, previous radiation on chest wall due to previous breast-conserving surgery, neoadjuvant chemotherapy due to locally advanced tumor stage, and skin-reducing mastectomy. Postoperative complications were defined as any of the following: major complications (i.e., active bleeding or wound dehiscence), minor complications (i.e., hematoma, seroma, surgical-site infection, <20% skin or nipple necrosis, and reoperation due to wound dehiscence), or any other complication.

Each patient is evaluated in a multidisciplinary council prior to surgery, which includes specialists from surgical oncologists, obstetrics and gynecology, genetics, and medical oncology. Based on the outcomes of the council, surgical recommendations are made for the patients. Patients were compared in terms of complications according to whether they previously received radiotherapy (RT) or neoadjuvant chemotherapy (CT). In addition, complication rates were compared between patients who underwent skin reduction and those who did not.

### 2.2. Data Collection and Statistical Analysis

General characteristics and clinicopathological patient data were collected retrospectively from the hospital data system. For data that could not be found, we completed the data by contacting the patients directly. Information on patient age, height, weight, and comorbid diseases was collected. Participants were screened for whether they had undergone breast-conserving surgery (due to breast cancer) before the genetic test result, thus creating a patient group that had previously received radiotherapy. Patients with locally advanced diseases at the time of diagnosis were identified and a patient group that received neoadjuvant chemotherapy was created. Patients who underwent skin-reducing mammoplasty due to breast volume were also grouped. The clinical and pathological stages of the patients were also collected and presented in the demographic data table.

Postoperative complications were divided into two groups as either major or minor complications. The group previously receiving radiotherapy, the group receiving neoadjuvant chemotherapy, and the group not receiving any neoadjuvant treatment were compared with each other, and the groups with and without skin-reducing mammoplasty were compared in terms of complication rates.

### 2.3. Surgical Technique

The surgeries were performed in two parts by the general surgery and gynecology and obstetrics teams under a single anesthesia period. Minimally invasive risk-reducing salpingo-oophorectomy was the preferred method for all patients and the surgeries were performed laparoscopically. Since all patients who were recommended for bilateral oophorectomy also requested a hysterectomy, they underwent laparoscopic total hysterectomy and bilateral salpingo-oophorectomy (TLH-BSO). All patients underwent bilateral skin-sparing mastectomy with implant reconstruction through a transverse incision extending from the areolar margin to the anterior axillary line. If skin reduction mastectomy was to be performed, a keyhole incision was preferred. Implants were placed subpectorally.

**Figure 1:**
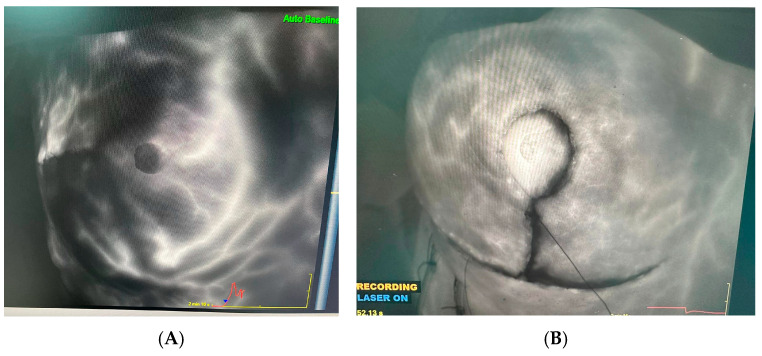
(**A**): Vascularization control with spy device after implant reconstruction with transverse incision. (**B**): Vascularization control with spy device in implant reconstruction after skin reduction mastectomy.

According to the literature, skin flaps should be thicker than 5 mm to prevent necrosis; therefore, when skin flaps were made, care was taken to ensure that the flap thickness was greater than 5 mm [13]. All patients who underwent bilateral skin-sparing mastectomy also received bilateral implants. During surgery, the vascularization of the skin flaps of all patients was confirmed intraoperatively with a SPY immunofluorescence device. The immunofluorescence technique is based on the principle of observing fluorescent antibodies bound to a certain molecule under light. The labeled antibodies brighten under light and show the location of the target molecule. In our surgeries, we use this device to show flap vascularization. After the patient is injected intravenously with indocyanine green, the flap is visualized with the SPY device; if there is a brightness, it is indicated that there is blood supply; if it is seen in black color, vascularization is impaired (Figure 1).

## 3. Results

### 3.1. General Features

The mean age was 44 ± 7.51 years for the group that previously received radiotherapy; 39.42 ± 8.14 years for the group that received neoadjuvant chemotherapy, and 44.74 ± 6.62 years for the patient group who did not receive any neoadjuvant treatment. When the groups were compared in terms of age, there was a significant difference between them due to the younger population of the group receiving neoadjuvant chemotherapy.

Detailed information about the patients’ ages, weights, tumor stage, and hormone receptor status is given in Table 1 and Table 2.

### 3.2. Surgical Technique

We performed skin-sparing mastectomy with implant reconstruction and TLH-BSO on all patients within the same anesthesia period. The mean duration of the entire procedure was 240 ± 52.37 (120–360) mins. The mean duration of skin-sparing mastectomy with implant reconstruction was 150.33 ± 53.75 (60–300) min and the mean duration of TLH-BSO was 75.55 ± 31.96 (30–150) min (Table 3).

The skin flap thickness was greater than 5 mm in all our patients. Flap vascularization was checked by SPY immunofluorescence and all patients with suspected vascularization were followed closely.

### 3.3. Complications

When the group that received breast-conserving surgery followed by radiotherapy before the genetic test result and the group that received neoadjuvant chemotherapy due to locally advanced disease were compared with the group that did not receive any neoadjuvant therapy, a statistically significant difference was found only in terms of seroma (*p* = 0.034). No seroma was observed in the patient group that did not receive any neoadjuvant therapy (Table 4).

Although there was a proportionally significant difference in the numbers of wound dehiscence, wound infection, skin necrosis, and re-operations due to dehiscence, this difference was not statistically significant. Wound dehiscence was observed in 57% of patients receiving neoadjuvant chemotherapy compared with 28.6% in the patient group who received preoperative radiotherapy and only 14.3% in those who did not receive neoadjuvant treatment.

**Figure 2:**
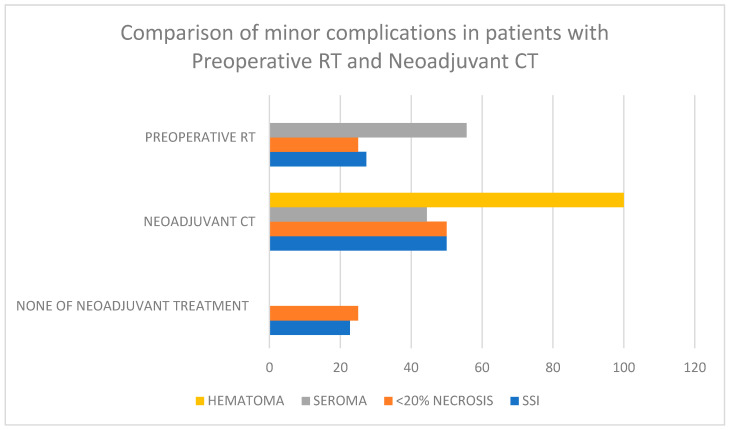
Comparison of minor complications in patients with Preoperative RT and Neoadjuvant CT.

Considering surgical-site infection, the rate was 50% in the neoadjuvant chemotherapy group while it was 27.3% and 22.7% in the groups receiving preoperative radiotherapy and those not receiving neoadjuvant treatment, respectively. The rate of wound necrosis was 50% in the neoadjuvant chemotherapy group and 25% in the other groups. Reoperation was mostly in the neoadjuvant chemotherapy group due to wound site dehiscence, but most of these were minor dehiscences that could only be repaired with primary suturing (Figure 2).

In total, 9 of 61 patients had undergone skin-reducing mastectomy. When these patients were compared in terms of minor and major complications, no statistically significant difference was observed. Although the percentage of complication rates in terms of surgical-site infection, wound necrosis, and wound dehiscence were higher in patients who underwent skin-reducing mastectomy, this rate was not statistically significant (Table 5).

## 4. Discussion

Due to the high-penetrance gene profile, the lifetime risk of developing cancer is significantly reduced in patients who undergo bilateral mastectomy and risk-reducing salfingo-oophorectomy [1,4,5].

In our clinic, 61 patients underwent skin-sparing mastectomy with implant reconstruction and risk-reducing salpingo-oophorectomy due to their high-penetrance gene profile. The patient’s age, BMI, and additional medical conditions that could impair the wound-healing process were noted. None of the patients were active smokers. Patients were grouped as those who received neoadjuvant treatment and those who underwent skin reduction procedures. Complication rates compared major and minor complications. Although there are few studies in the literature where these surgeries are performed in a single session, these surgeries are generally performed in two separate anesthesia sessions [4,14]. Our study represents one of the largest series of breast and gynecologic surgeries performed in the same anesthesia period for patients with a high-penetrance gene profile.

While complication rates in the literature are between 20 and 40%, our total complication rate was 47% [14,15,16]. It is important to underline that most of these complications were minor. According to Table 4, the only statistically significant value was seroma (*p*: 0.034) Although there was no statistically significant difference in wound dehiscence, this rate was higher in the group receiving Preop RT and neoadjuvant CT. We think that the limited number of patients was the biggest obstacle in the absence of a statistically significant difference. Surgical-site infection was seen in 50% of the group receiving neoadjuvant CT. While there are studies in the literature indicating that chemotherapy has no effect on wound healing, this rate was in favor of the opposite in our study [8,9,17]. The rate of skin necrosis was also higher in the neoadjuvant CT group (50%). Although 13 of our patients required a second surgery, most of these procedures were simple primary wound closures. In our clinic, hyperbaric oxygen therapy can be given to patients with skin necrosis if deemed necessary. This allows necrotic wounds to be followed without major dehiscence. In addition, we reduce our wound necrosis rates by confirming skin vascularization with the SPY immunofluorescence device we use intraoperatively. Patients with suspected vascularization in the skin flaps after intraoperative SPY immunofluorescence confirmation were closely followed up and, if necessary, early hyperbaric oxygen therapy was initiated. Thus, all patients could be followed without major wound dehiscence, surgical-site infections, or implant loss. Implant loss occurred in one patient due to wound dehiscence and they were in the group that did not receive any neoadjuvant treatment and used steroids due to their rheumatological disease. Afterwards, a latissimus dorsi flap was applied successfully to this patient.

Another important factor in our ability to treat wounds without dehiscence was the experienced wound care team in our clinic. To evaluate possible necrosis in the tissue in advance, tissue perfusion can be assessed with intraoperative indocyanine green [18]. Early recognition of necrosis in the postoperative period is particularly important. In this process, the tissue can be healed with wound care products with assistance from wound care nurses and teams. When discoloration of the nipple is noticed in the early period, it might be preferable to loosen the sutures and use hydropolymer wound dressings that provide optimum moisture and heat balance in the tissue. In advanced necrosis, instead of aggressive surgical debridement, the aim should be to dissolve and remove necrosis and eschar tissue with collagenase-containing creams. Simultaneously with the removal of the eschar tissue, healing of the nipple can be achieved with hyaluronic acid-containing creams that support tissue epithelialization. The wound care team from our clinic provided treatment to patients in the risk group with these methods.

Bilateral skin-sparing mastectomy and implant can be performed in our clinic within the same surgery period of bilateral mastectomy that is recommended in the literature. Gynecological surgery times are also similar to what is reported in the literature. Although our surgery times are slightly below the recommended times in the literature, performing breast and gynecological surgeries in the same anesthesia session also helps reduce surgical-site infections [4]. While surgical-site infection rates in implant-based breast surgery are up to 43% in the literature, our surgical-site infection rate was considerably lower at 36% [17,19,20].

Current publications in the literature say that neoadjuvant chemotherapy has no effect on wound healing [9]. A study conducted on 44,000 patients showed that neoadjuvant chemotherapy had no effect on wound healing but, implant-based reconstructions were excluded from the study [8]. Our study is one of the few studies investigating the effect of neoadjuvant chemotherapy on complications in implant-based breast surgery that found no effect on wound healing, which is consistent with other studies [8,9,17].

Studies have reported an increase in minor complications after skin-reducing mastectomy and implant application. Others have shown no major differences in terms of major complications and implant loss [21,22]. In our study, performing a skin-reducing mastectomy did not pose a significant risk in terms of major or minor complications. Although the percentage of wound dehiscence, necrosis, and surgical-site infections was higher this difference was not high enough to make a statistical difference. Our low complication rates were due to the SPY immunofluorescence device used intraoperatively and the fact that a single experienced team performed all of the surgeries.

This study derives its power from being one of the largest studies investigating the complication rates in patients who underwent skin-sparing mastectomy with implant reconstruction due to a high-penetrance gene profile. It is also the only study to include a group that previously received radiotherapy due to breast-conserving surgery. It is thought that the difference will be statistically significant in larger patient series. Although no financial analysis was performed, it is clear that a single hospital stay for two surgical procedures combined with a single anesthesia session would provide savings for the institution when compared with two hospital stays for two separate surgical procedures.

## 5. Conclusions

Due to the high-penetrance gene profile, bilateral skin-sparing mastectomy and salfingo-oophorectomy are safely used surgical methods. In patients with this profile, performing two surgeries during the same anesthesia session is a viable option. Intraoperative SPY immunofluorescence usage, close postoperative follow-up, and hyperbaric oxygen therapy (if necessary) can reduce the complications of surgery. The advantage of our study is that it is a single-center, multidisciplinary team effort. However, the number of cases in the control group is limited. We think that the difference will be statistically significant with the new patients we will involve in our study. Prospective studies with longer follow-up periods are needed.

## Figures and Tables

**Table 1:** Demographic data based on gene profiles.

			BRCA1 (*n*: 24)	BRCA2 (*n*: 28)	Other High Penetrant Gene Group (*n*: 9)
Age			42.28 ± 7.82 (30–58)		
BMI			25.08 ± 4.19 (17.3–39.1)		
Surgery Duration, min			150.33 ± 53.75 (60–300)		
Relative Comorbidity	No		21	21	9
			(87.5%)	(75%)	(100%)
	Yes		2	4	0
			(8.33%)	(14.28%)	(0%)
Hormone Receptor Expression; (*n*: %)	ER (+)		6	15	5
			(25%)	(53.57%)	(55.5%)
	PR (+)		6	13	3
			(25%)	(46.42%)	(33.33%)
HER2 (*n*: %)	HER2 (+)		2	3	2
			(8.33%)	(10.71%)	(22.22%)
	HER2 (−)		21	22	7
			(87.5%)	(78.57%)	(77.77%)
Previously RT (*n*: %)	No		20	16	7
			(83.33%)	(57.14%)	(77.77%)
	Yes		3	9	2
			(12.5%)	(32.14%)	(22.22%)
Neoadjuvant CT (*n*: %)	No		10	19	4
			(41.66%)	(67.85%)	(44.44%)
	Yes		13	6	5
			(54.16%)	(21.42%)	(55.55%)
Clinical Stage; (*n*: %)	T	T1	9 (37.5%)	9 (32.14%)	3 (33.33%)
		T2	8 (33.33%)	9 (32.14%)	4 (44.44%)
		T3	1 (4.16%)	0 (0%)	0 (0%)
	N	N0	7 (29.16%)	8 (28.57%)	5 (55.55%)
		N1	1 (4.16%)	2 (7.14%)	1 (11.11%)
		N2	8 (33.33%)	8 (28.57%)	1 (11.11%)
		N3	2 (8.33%)	0 (0%)	0 (0%)
Pathologycal Stage; (*n*: %)	T	T0	9 (37.5%)	3 (10.71%)	2 (22.22%)
		T1	7 (29.16%)	8 (28.57%)	3 (33.33%)
		T2	2 (8.33%)	6 (21.42%)	2 (22.22%)
		T3	1 (4.16%)	0 (0%)	0 (0%)
	N	N0	7 (29.16%)	9 (32.14%)	5 (55.55%)
		N1	1 (4.16%)	2 (7.14%)	1 (11.11%)
		N2	8 (33.33%)	8 (28.57%)	1 (11.11%)
		N3	2 (8.33%)	0 (0%)	0 (0%)
Skin-Reducing Mastectomy; (*n*: %)			4	4	1
			(16.66%)	(14.28%)	(11.11%)

BMI: body mass index; ER: estrogen receptor; PR: progesteron receptor; Previously RT: previously received radiotherapy; Neoadjuvant CT: neoadjuvant chemotherapy.

**Table 2:** Distribution of demographic and clinical characteristics of patients according to neoadjuvant treatment characteristics.

	Groups (*n*, %)			*p* Value
	Previousl RT (16, 26.2%)	Neoadjuvant CT (26, 42.6%)	Not Receiving Any Neoadjuvant Treatment (19, 31.2%)	
Age, Years, Mean ± SD	44 ± 7.51	39.42 ± 8.14	44.74 ± 6.62	0.044 ^F^
BMI, Median, Distribution	24.25 (20.8–38.8)	23.9 (19.2–39.1)	25 (17.3–32)	0.711 ^H^
Gene Profile, *n* (%)				0.098 ^X2^
BRCA1	3 (12.5%)	14 (58.3%)	7 (29.2%)	
BRCA2	11 (39.3%)	7 (25%)	10 (35.7%)	
Other High Penetrant	2 (22.2%)	5 (55.6%)	2 (22.2%)	
Nipple Ssparing Mastectomy, *n* (%)				0.846 ^X2^
No	2 (20%)	5 (50%)	3 (30%)	
Yes	14 (27.5%)	21 (41.2%)	16 (31.3%)	
Skin Reducing Mastectomy, *n* (%)				0.270 ^X2^
No	15 (28.8%)	20 (38.5%)	17 (32.7%)	
Yes	1 (11.1%)	6 (66.7%)	2 (22.2%)	
Comorbidities *, *n* (%)				0.569 ^X2^
No	14 (25.9%)	22 (40.8%)	18 (33.3%)	
Yes	2 (28.6%)	4 (57.1%)	1 (14.3%)	
Skin-Sparing Mastectomy With Implant Reconstruction Surgery Time, min, median, Distribution	160 (90–240)	125 (60–180)	180 (80–300)	0.089 ^H^

^H^, Kruskal–Wallis test; ^F^, oneway Anova test; ^X2^, Pearson chi-square test; * comorbidities: diabetus mellitus and medical conditions requiring the use of steroids.

**Table 3:** Surgery time.

	Total (*n*: =61)
Mean Operative Time SSMI + TLH + BSO	240 ± 52.37 (120–360)
Mean Operative Time SSMI	150.33 ± 53.75 (60–300)
Mean Operative Time TLH + BSO	75.55 ± 31.96 (30–150)

SSMI: Skin-sparing mastectomy with implant reconstruction. TLH + BSO: total laparoscopic hyhystesterectomy + bilateral salhingoophorectomy.

**Table 4:** Comparison of preoperative RT and neoadjuvant CT complications.

		Preoperative RT (16, 26.2%)	Neoadjuvant CT (26, 42.6%)	None of Neoadjuvant Treatment (19, 31.2%)	*p*
Major Complications; *n* (%)	Active Bleeding				0.504 ^X2^
	No	16 (26.7%)	25 (41.7%)	19 (31.6%)	
	Yes	0 (0%)	1 (100%)	0 (0%)	
	Wound Dehiscence				0.273 ^X2^
	No	12 (25.5%)	18 (38.3%)	17 (36.2%)	
	Yes	4 (28.6%)	8 (57.1%)	2 (14.3%)	
Minor Complicaitons; *n* (%)	SSI				0.539 ^X2^
	No	10 (25.6%)	15 (38.5%)	14 (35.9%)	
	Yes	6 (27.3%)	11 (50%)	5 (22.7%)	
	<20% Skin Necrosis				0.759 ^X2^
	No	12 (26.7%)	18 (40%)	15 (33.3%)	
	Yes	4 (25%)	8 (50%)	4 (25%)	
	Seroma				0.034 ^X2^
	No	11 (21.2%)	22 (42.3%)	19 (36.5%)	
	Yes	5 (55.6%)	4 (44.4%)	0 (0%)	
	Hematoma				0.249 ^X2^
	No	16 (27.1%)	24 (40.7%)	19 (32.2%)	
	Yes	0 (0%)	2 (100%)	0 (0%)	
Reoperation; *n* (%)	No	12 (25%)	19 (39.6%)	17 (35.4%)	0.380 ^X2^
	Yes	4 (30.8%)	7 (53.8%)	2 (15.4%)	

^X2^, Pearson chi-square test. SSI: surgical-site infection.

**Table 5:** Comparison of complications in skin-reducing mastectomy surgery.

		Skin Reducing Mammoplasty		*p*
		Yes (*n*: 9)	No (*n*: 52)	
Major Complications; *n* (%)	Active Bleeding	0 (0%)	1 (1.92%)	0.675
	Wound Dehiscence	3 (33.33%)	11 (21.15%)	0.412
Minor Complications; *n* (%)	SSI	4 (44.44%)	18 (34.61%)	0.571
	<20% Skin Necrosis	3 (33.33%)	13 (25%)	0.6
	Seroma	1 (11.11%)	8 (15.38%)	0.339
	Hematoma	0 (0%)	2 (3.84%)	0.55
Reoperation; *n* (%)	Due to Wound Dehiscence or Other Complications	1 (11.11%)	10 (19.23%)	0.682

## Data Availability

Data are available from the corresponding author upon reasonable request.

## Abbreviations

The following abbreviations are used in this manuscript:

RT	Radiotherapy
CT	Chemotherapy
RRSO	Risk-reducing salpingo-oophorectomy
RRBM	Risk-reducing bilateral mastectomy
NCCN	National Comprehensive Cancer Network
NACT	Neoadjuvant chemotherapy
SSMI	Skin-sparing mastectomy with implant reconstruction
TLH	Total laparoscopic hysterectomy
BSO	Bilateral salpingo-oophorectomy
BMI	Body mass index
ER	Estrogen receptor
PR	Progesteron receptor
